# 

**DOI:** 10.1192/bjb.2024.122

**Published:** 2025-08

**Authors:** Aysha Zabin, Sumeet Gupta

**Affiliations:** Speciality doctor with Tees, Esk and Wear Valleys NHS Foundation Trust, Harrogate, UK; Consultant psychiatrist with Tees, Esk and Wear Valleys NHS Foundation Trust, Harrogate, UK. Email: sumeetgupta_2000@yahoo.com

**Figure:**
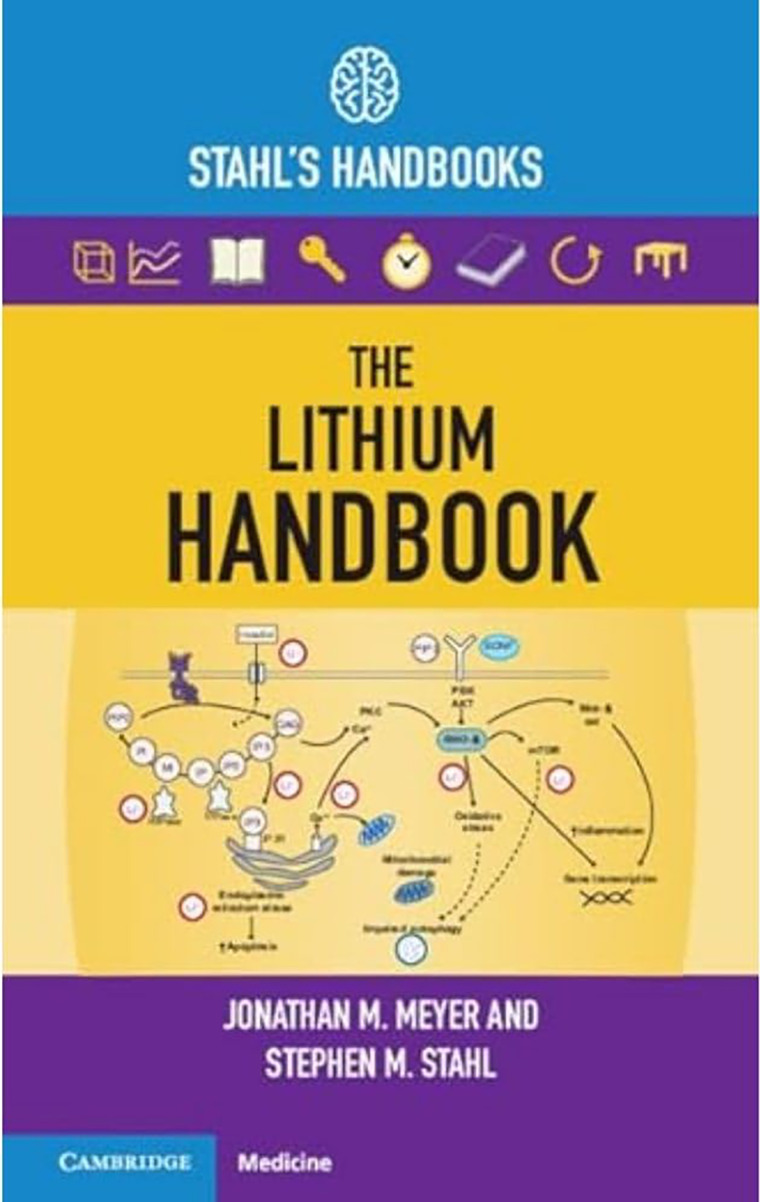

Despite increasing evidence supporting lithium's superiority over other mood stabilisers in the treatment of bipolar disorder, prescribers continue to favour alternatives such as atypical antipsychotics. Several factors contribute to this discrepancy, the most significant being the perception of lithium as a toxic drug with potentially severe side-effects that require close monitoring. Although clinical guidelines and expert recommendations consistently advocate its use, these have not significantly changed its underutilisation. There was a need for a definitive, evidence-based resource that could reassure prescribers, correct misconceptions and ensure that patients do not miss out on this highly effective treatment.

The latest handbook by renowned psychopharmacologists Jonathan Meyer and Stephen Stahl offers the most current literature on lithium in an accessible format. It provides valuable insights into lithium's effectiveness in treating various psychiatric conditions and its adverse effects. It also emphasises that risks often associated with lithium, such as renal problems, are frequently exaggerated and can be effectively managed without discontinuing its use.

The book delves into the complexities of lithium prescribing in a reader-friendly way. It incorporates high-quality evidence, both primary and secondary, to substantiate the facts and findings. Beyond lithium, the book explores the management of bipolar disorder, offering recent research evidence. Structured in eight chapters, it covers efficacy, renal handling, pharmacokinetics, initiation and monitoring, common adverse effects, toxicity, special populations and discontinuation. Each chapter includes clear headings and subheadings that improve the reading experience by providing a smooth flow of information. A ‘what to know’ section under each subheading summarises concisely, and each chapter ends with key points. Tables, ‘in-depth’ boxes and information boxes further guide readers through each topic.

The book provides helpful guidance regarding managing lithium's adverse effects and emphasises that most can be effectively managed without discontinuation. The suggestions of using amiloride or acetazolamide to treat diabetes insipidus, cinacalcet for hypercalcaemia and primidone or topiramate for tremors would also reinforce effective management of adverse effects.

All referenced studies are appropriately cited, with a critical analysis of their findings, giving the volume a reference book feel. It can also serve as a practical tool for daily use, answering any questions related to lithium and earning it a ‘companion’ status for clinicians. Even though some chapters repeat certain points, these repetitions help clarify the subject matter. Therefore, *The Lithium Handbook* is an essential addition to the toolkit of all mental health clinicians.

